# Oxford Hip and Shoulder Scores as Potential Tools for the Early Detection of Avascular Necrosis in Apparently Unaffected Sites in Sickle Cell Disease: Results from a Prospective Cohort Study

**DOI:** 10.3390/jcm14165744

**Published:** 2025-08-14

**Authors:** Maddalena Casale, Giuseppe Toro, Federica Porcelli, Vincenzo Spadola, Rosamaria Rosso, Anna Bulla, Alessandra Quota, Concetta Simona Perrotta, Saveria Campisi, Francesco Arcioni, Maria Maddalena Marrapodi, Silverio Perrotta

**Affiliations:** 1 Department of the Woman, The Child and General and Specialized Surgery, University of Campania Luigi Vanvitelli, 80138 Naples, Italy; federica.porcelli@studenti.unicampania.it (F.P.); mariamaddalena.marrapodi@unicampania.it (M.M.M.); silverio.perrotta@unicampania.it (S.P.); 2 Department of Medical and Surgical Specialties and Dentistry, University of Campania Luigi Vanvitelli, 80138 Naples, Italy; giuseppe.toro@unicampania.it; 3 Thalassemia Centre, Hospital “Giovanni Paolo II”, 97100 Ragusa, Italy; vincenzo.spadola@asp.rg.it; 4 Thalassemia Unit, AOU Policlinico G.Rodolico-San Marco, 95123 Catania, Italy; rosellinarosso@gmail.com (R.R.); anna.bulla24@gmail.com (A.B.); 5 Thalassemia Centre, Hospital Vittorio Emanuele, 93012 Gela, Italy; alequota@hotmail.com (A.Q.); kettyperrotta@yahoo.it (C.S.P.); 6 Thalassemia Unit, Hospital “Umberto Primo”, 96100 Siracusa, Italy; campisisaveria61@gmail.com; 7 Pediatric Oncology-Hematology, Hospital of Perugia, 06122 Perugia, Italy; francesco.arcioni@ospedale.perugia.it

**Keywords:** avascular necrosis, hip, osteonecrosis, oxford hip score, oxford shoulder score, patient-reported outcome measures, shoulder, sickle cell disease

## Abstract

**Objectives:** Sickle Cell Disease (SCD) has a significant impact on the musculoskeletal system. The use of the Oxford Hip (OHS) and Shoulder score (OSS) as patient-reported outcome measures (PROMs) revealed a high rate of long-term impairment in joints with a pre-existing diagnosis of avascular necrosis (AVN). With this study, we aimed at detecting dysfunction in joints apparently not affected by AVN. **Methods:** This is a subgroup analysis of a previous core study assessing the OHS and OSS in 47 SCD patients with a pre-existing diagnosis of AVN. For this study, only patients with a pre-existing diagnosis of isolated AVN (only hip or only shoulder) were selected, and the OHS or OSS was measured in previously unaffected joints. **Results:** Among 37 patients with isolated AVN, 19 (51%) patients presented abnormal scores in the apparently unaffected joint; 16 (50%) patients with pre-existing isolated hip AVN had an abnormal OSS; and 9 (56%) had moderate to severe shoulder impairment. All patients with pre-existing isolated shoulder AVN had an abnormal OHS, with severe hip impairment in two out of three. As per clinical practice, patients with an abnormal score were prescribed joint magnetic resonance imaging (MRI) and/or X-rays. Only 10 out 19 (53%) performed imaging studies and all showed signs of AVN. **Conclusions:** Abnormal OHS and OSS values indicated a high rate of joint dysfunction in sites apparently not affected by AVN. The routine use of these PROMs should be applied to all SCD adults and subjects with a pathological score should have priority access to diagnostic radiological tests.

## 1. Introduction

Sickle cell disease is an autosomal recessive disorder causing the production of the abnormal haemoglobin S (HbS). In the deoxygenated state, HbS polymerizes and disrupts the architecture and flexibility of red blood cells, promoting cellular dehydration and oxidative stress, with the final results of haemolytic anaemia and vaso-occlusion with ischaemia-reperfusion injury [1,2].

Although SCD is included in the group of rare diseases in European countries, the WHO has defined the disease as a global health problem due to the increase in the number of new diagnoses in all countries with a low prevalence of the disease historically [3,4].

Osteonecrosis or avascular necrosis (AVN) is a complication that can also be identified in patients with other clinical conditions, such as systemic steroid administration, habitual alcohol use, and antiphospholipid syndrome [5]. It is a highly prevalent complication in patients with sickle cell disease (SCD), with rates as high as 50% even in young adults (35 years) [6]. Moreover, endocrine disease, osteoporosis, and low bone marrow density should be recognized as risk factors for AVN in SCD [7]. Since osteonecrosis is a serious, potentially disabling, and progressive complication, its development is an indication for a bone marrow transplant (BMT) from a matched sibling donor or matched unrelated donor, if available [8,9]. However, the effect of BMT on the onset and progression of bone lesions is controversial [10]; therefore, interventions aimed at the early diagnosis of AVN are crucial to reducing the number of patients affected by this progressive complication, which becomes irreversible in advanced stages [11].

These findings underline the importance of including the regular monitoring of joint functions in health care maintenance programs for SCD, especially for the hip and shoulder functions, which are the sites most affected by sickle-related osteonecrosis, causing disability, impaired working capability, chronic pain, and reduced quality of life [12,13].

Unfortunately, even today there is no standardized method for monitoring joint complications in SCD, with the final result being that many patients are diagnosed at an advanced stage of the disease, with very poor outcomes of therapeutic interventions [14].

We used the Oxford hip score (OHS) and Oxford Shoulder Score (OSS) to evaluate the long-term outcome of SCD patients with a pre-existing diagnosis of AVN [15]. The Oxford scores are tools validated in several languages, used in numerous national and international registries and in various clinical settings [16,17,18]. They evaluate various domains, such as pain, motor skills, and the impact on daily activities, and they are particularly easy to handle due to the brevity (12 questions), the simple calculation of the final score, and the clear correlation between the score and the clinical and therapeutic actions to be performed, so they can be easily used and interpreted even by non-specialized personnel [15].

In our previous study, as many as 61% and 68% patients with pre-existing hip and shoulder AVN reported pathological OHS and OSS values respectively, indicating a significant long-term burden in the joint site affected by AVN [15].

With this study, we aimed to evaluate the function of multiple joints in patients with SCD previously diagnosed with isolated AVN of the hips or shoulders, measuring the Oxford score in the apparently undamaged joint sites and assessing the results of joint magnetic resonance imaging (MRI) and/or X-rays.

## 2. Methods

This is a subgroup analysis of a previous multicenter prospective study on the long-term outcomes of pre-existing AVN in patients with SCD using the OHS and OSS. In the core study, a pre-existing diagnosis of AVN was reported in a total of 47 (13%) subjects among 357 SCD patients actively followed at six clinical centres. All patients completed PROMs (OHS and OSS), and the following data were collected: sex, age, genotype, site of AVN, age at diagnosis of AVN, laboratory values, conventional therapies, and splenectomy [15].

**Figure 1:**
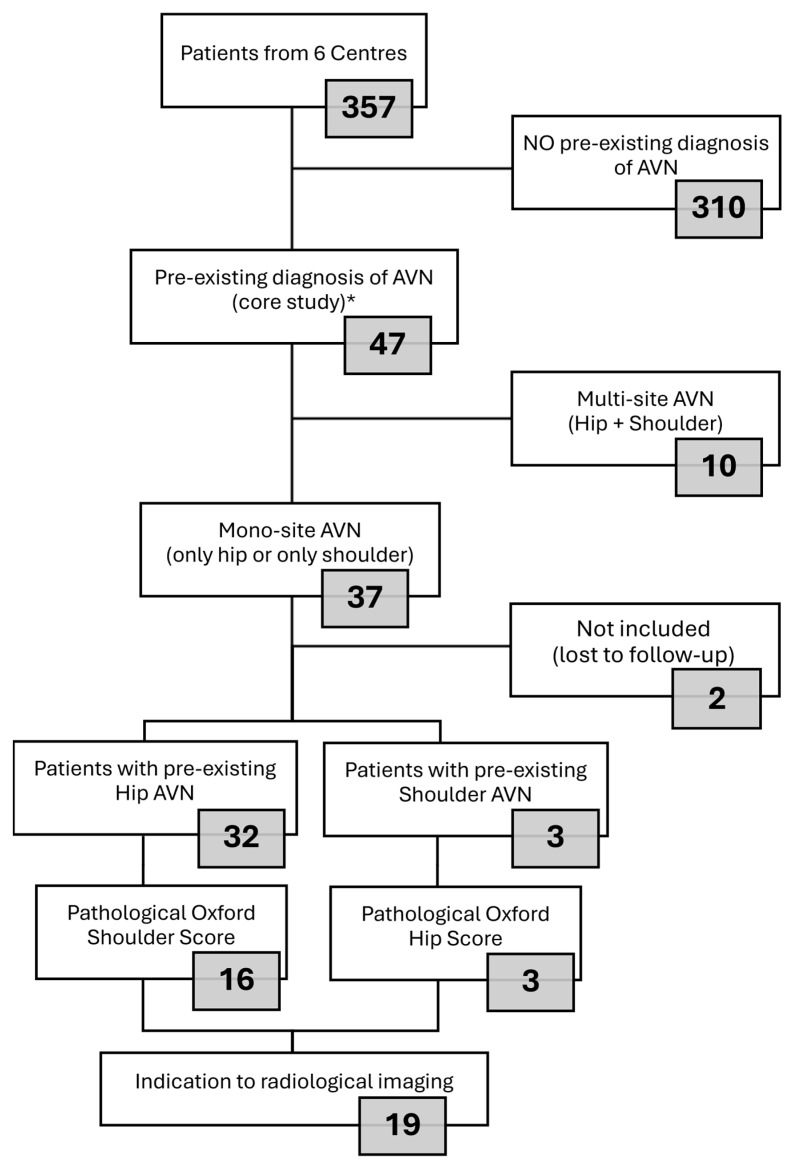
Patient selection. AVN: avascular necrosis. * Core study [15].

Multisite AVN (hip + shoulder) was reported in 10 (22%) patients, and these patients were excluded from the present analysis, which aimed to assess function and pain in unaffected joints. Therefore, we selected patients with mono-site AVN (only hip or only shoulder) to assess the score of PROMs in the joints apparently unaffected by AVN. Figure 1 shows the patient selection for enrolment in the present subgroup analysis.

The grading for both scores (OHS and OSS) was as follows: score 40–48 = satisfactory joint function; score 30–39 = mild to moderate impairment; score 20–29 = moderate to severe impairment; score 0–19 = severe impairment.

According to clinical practice, patients with a pathological score (≤39) in a previously unaffected joint site were prescribed bilateral joint MRI and/or X-rays if needed. The reporting was performed locally at each clinical center and the images were centrally evaluated by a single orthopedist for the assessment of Ficat classification into stage 0 to stage 4 for the Hip, and the Cruess classification into Stages I to IV was used for the shoulder [17,18].

The diagnosis of AVN was performed though MRI using the criteria recommended by Ge H. et al. [19]. For the Ficat and Cruess classification systems, we modified the staging criteria to align with MRI findings, following established adaptation guidelines [20,21,22,23]. In doubtful cases, the classification was accomplished through a contemporary evaluation of X-rays performed in anteroposterior and in “frog-leg” views for the hip, and anteroposterior, axillary, and rotational radiographs were performed for the shoulder.

The research protocol was approved by the local ethical committees and was implemented in accordance with the Declaration of Helsinki and the ICH guidelines for good clinical practice. All patients received verbal and written explanation of the aims and procedure of the study, and written informed consent was obtained.

We used the same approach to statistically analyze the data as in the core study [15], and IBM SPSS Statistics v28 software was used.

Categorical variables and quantitative variables were analyzed using the frequency and percentage (*n*, %) and the central tendency indexes and variability, respectively. A descriptive statistical analysis was also performed for patients with hip AVN and with shoulder AVN. As SCD is a rare disease, the number of patients evaluated was small, and we used non-parametric tests, as the use of parametric tests would make the analysis substantially unreliable and therefore inapplicable.

## 3. Results

According to the inclusion and exclusion criteria, 19 out 37 (51%) patients with isolated AVN enrolled in the core study [15] presented a pathological score in the unaffected joint; 16 patients had pre-existing isolated hip AVN and an abnormal OSS; and 3 patients had pre-existing isolated shoulder AVN and abnormal OHS. In the present subgroup analysis, we considered 35 patients, as 2 subjects enrolled in the core study and affected by mono-site AVN temporarily moved to other cities for work reasons at the time of this subgroup analysis (Figure 1).

The sample included 7 (37%) males and 12 (63%) females of a mean age of 46.47 (±13.50) at the last follow up. The mean age at SCD diagnosis was 7.9 years (±11.94), and at AVN diagnosis it was 35.58 (±13.01). The patients were actively followed at the treatment centers for a mean post-AVN follow-up time of 10.11 (±6.18) and a total follow-up of 37.95 (±17.79) years. The characteristics of the study population are shown in Table 1.

**Figure 2:**
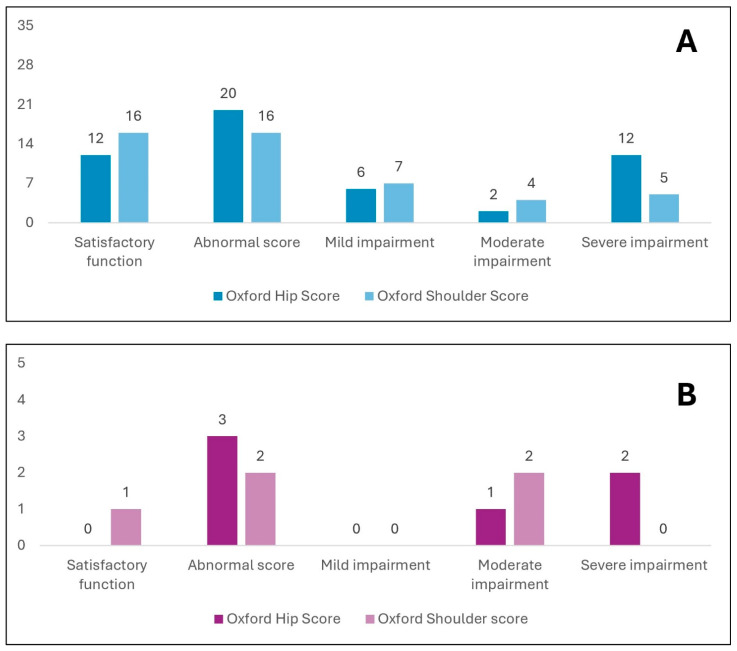
Oxford Hip Score and Oxford Shoulder Score in patients with a preexisting diagnosis of isolated Hip avascular necrosis (AVN) (**A**) and isolated Shoulder AVN (**B**). AVN: avascular necrosis.

Figure 2 reports the Oxford scores (hip and shoulder) in patients with a pre-existing diagnosis of isolated AVN from the study core population.

Panel A reports the Oxford hip and shoulder score in patients with a pre-existing diagnosis of isolated hip AVN (32 patients), showing that 20 patients (61%) had limitations in hip joint function, and among them, 12 (37%) had a score in the severe impairment range. In the absence of a pre-existing diagnosis of shoulder AVN, 16 (50%) of patients with hip AVN had an impaired Oxford shoulder score, and more than half of patients had moderate to severe impairment. Panel B reports the scores in patients diagnosed with shoulder AVN only; two out of three had shoulder score values indicative of moderate impairment and all three patients had altered hip scores, with severe hip impairment in two out of three.

Finally, all patients who presented an altered score in a site without a diagnosis of AVN were prescribed a diagnostic radiological assessment, as per clinical practice.

Nine patients had not yet undergone joint MRI at the time of data collection for this study due to organizational problems.

**Figure 3:**
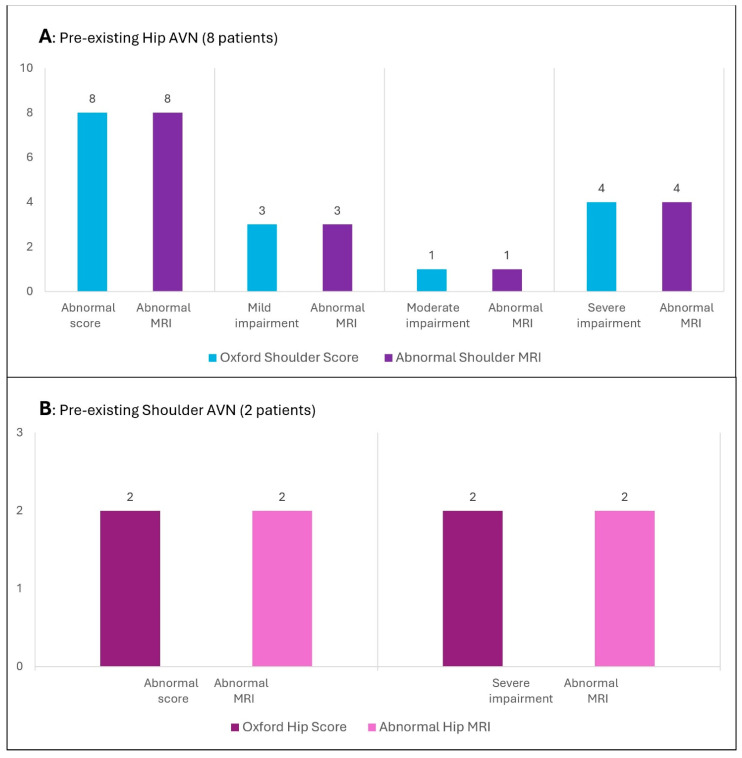
Oxford Score and MRI results in patients with a preexisting diagnosis of isolated hip AVN (**A**) and isolated shoulder AVN (**B**). MRI: magnetic resonance imaging. Satisfactory function ≥ 40; abnormal score < 40; mild–moderate impairment 30–39; moderate–severe impairment 20–29; severe impairment < 19. AVN: avascular necrosis.

Therefore, MRI images of 10 subjects were evaluated, as well as of 8 subjects with pre-existing hip AVN and an altered OSS (shoulder MRI) and 2 patients with pre-existing shoulder AVN and an abnormal OHS (hip MRI). The MRI results from patients with pathological Oxford scores were all abnormal (Figure 3).

**Figure 4:**
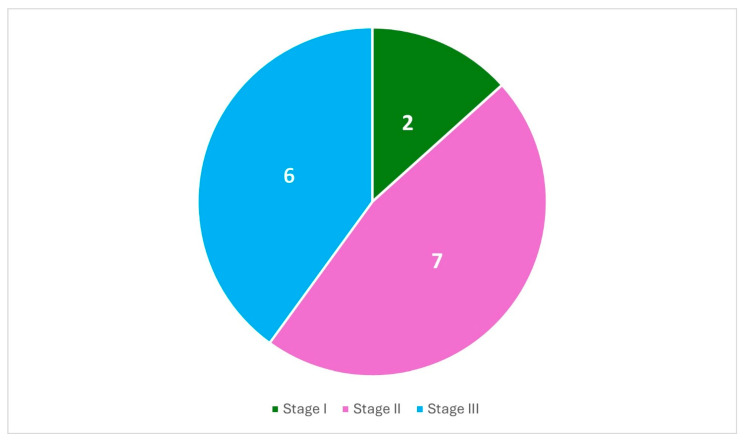
Cruess Classification in patients with abnormal Oxford shoulder scores and pre-existing isolated hip AVN.

The results of the shoulder MRI performed in 15 joints according to the Cruess classification are shown in Figure 4.

Hip MRI was performed in three sites: one patient underwent bilateral MRI, one patient underwent unilateral MRI, and one site was classified into stage 1 and 2 sites into stage 2, according to Ficat classification.

## 4. Discussion

Osteonecrosis is a highly prevalent complication in patients with SCD. It has been reported that the diagnosis can remain unrecognized for months or years as joint pain, which is the initial and major onset symptom for this complication, can be intermittent; therefore, the patient may not report it during the clinical visits or classify it as the typical vaso-occlusive sickle-related pain [14].

Thanks to the use of joint-specific PROMs, the OHS, and the OSS, the present study reports that as many as 52% patients with a pre-existing diagnosis of isolated hip AVN also had shoulder impairment and 100% of subjects diagnosed with isolated shoulder AVN also had hip impairment, even in the absence of specific joint symptoms reported by the patients; therefore, the rate of multifocal forms (hip + shoulder) of AVN increased from 21%, as reported in the core study [15], to 68%, as observed in this subgroup analysis.

Moreover, the joint pathology in SCD is mostly bilateral, multisite, systemic, and not isolated to a single site; this should be taken into consideration when planning radiological imaging for suspected AVN in a joint site.

Indeed, in patients with SCD, osteoarticular pain is very frequent, nonspecific, diffuse, and multifocal, and it is the first and most frequent cause of admission for acute events [24,25,26]; therefore, joint-specific PROMs are necessary to identify joint AVN, preserve joint function, and improve quality of life through specific interventions [13].

All patients with an abnormal OHS or OSS score were prescribed a diagnostic radiological assessment, and all showed a relevant joint lesion, indicating the potential predictive value of the Oxford scores in subjects with SCD.

This is a finding of considerable interest and value as it demonstrates that a simple and cost-effective tool allows for a rapid and reliable screening of joint disorders in SCD and ensures the appropriateness of magnetic resonance imaging (MRI) requests, thus reducing costs for healthcare systems and the burden of disease management for physicians, patients, and families [27] and improving the detection of serious, progressive, and potentially disabling complications.

It has been reported that patients with SCD have limited access to some fundamental examinations for the management of the disease, such as transcranial Doppler for the primary prevention of cerebral stroke, and the recognition of such critical issues is necessary to implement actions that improve the management of the disease [28,29].

In our study, approximately half of the patients with an altered Oxford score and clinical indication of joint MRI did not have rapid access to the radiological examination due to organizational issues, and this highlights a further critical issue in the management of sickle-related complications and patient access to necessary diagnostic tests.

Indeed, among the factors limiting the access to advanced radiological examination, there are organizational problems, such as long waiting lists, the crowding of radiology services, often focused on the management of acute patients, the low priority for patients with chronic conditions, and a lack of knowledge about the potential for disability and progression caused by some complications in patients with chronic diseases [30].

The use of a simple tool that is able to evaluate the severity of joint dysfunction could help in the detection of joint complications in SCD and in the attribution of a priority code for access to advanced radiological examinations.

Interestingly, a high degree of avascular necrosis was also undetected before our evaluation using the Oxford scores, and most of them were classified into advanced stages, which generally require a surgical treatment to improve patients’ outcomes, and their early detection could be of aid [31,32]. Considering the low overall sensitivity for early-stage AVN (approximately 41%), radiograph-based detection should not be enough to identify AVN, especially in pre-collapse stages [33]. However, plain radiographs still play their role when planning a surgical procedure. Therefore, in our opinion, after detecting grade 3 AVN, an X-ray should be obtained. Anyway, no clear recommendations are currently available in SCD in both shoulder and hip AVN because of a lack of evidence [34,35,36]. Unfortunately, both shoulder and hip AVN in SCD are generally associated with worse outcomes, compared to patients without SCD, regardless the type of treatment [15].

So, AVN is a frequent, early, progressive, and worsening complication in SCD.

The clinical progression of asymptomatic hip AVN, detected just at radiological imaging due to a contralateral symptomatic osteonecrosis, was reported in 91% of patients, and as many as 77% of them had collapse of the femoral head with a positive correlation between the speed of progression and the severity of the radiological stage. Among patients with collapse, 75% required surgery for intractable pain, and the burden of surgery in patients with SCD, due to the pre-surgery preparation, the risk of anaesthesia, and the surgery itself, is a critical issue in the management of this complication.

The rates of progression reported for non-sickle-related AVN, such as those due to alcohol abuse, steroid use, systemic lupus erythematosus, or idiopathy, are significantly lower, from 7 to 33% compared to 91% reported in SCD [37].

These data point out the need for the early diagnosis of AVN. The main problem, reported in several reviews and meta-analyses on the topic, is the lack of a standardized measurable tool for evaluating joint dysfunction [38].

The reliability and validity of Oxford hip and shoulder scores have been assessed in different prospective studies; therefore, they are validated joint-specific PROMs in different languages (English, French, German, Italian, Dutch, Japanese) and used in cohort studies, audits, national joint replacement registries, and also in non-surgical clinical settings. As these scores are simple, cost effective, easy to use, and self-administered, they seem the most appropriate tools for the regular monitoring of joint function in clinical centers caring for patients with SCD [16,17,18].

Our study has some limitations, such as the small number of participants, the lack of data on possible other risk factors that would have refined the assessment, and the lack of MRI in all selected patients for practical organizational reasons.

In conclusion, our study demonstrated that the use of OHS and OSS is effective in identifying joint dysfunction in patients with SCD and might have significant predictive value regarding pathological alterations on MRI. We recommend the routine use of these PROMs in all adult patients with SCD, regardless of joint symptoms, and the performance of a radiological examination in the case of a pathological score.

## Figures and Tables

**Table 1:** Characteristics of the study population.

	*n* (%) or Mean (SD)
Patients with pre-existing isolated hip AVN	16 (84)
Patients with pre-existing isolated shoulder AVN	3 (100)
Male	7 (37)
Female	12 (63)
Genotype Sbeta°	10 (53)
Genotype Sbeta+	4 (21)
Genotype SS	5 (26)
Patients on HU	14 (74)
Splenectomy or splenic atrophy	12 (63)
Age at SCD diagnosis (years)	7.95 (±11.94)
Age at diagnosis of AVN (years)	35.58 (±13.01)
Age at last follow-up (years)	46.47 (±13.50)
Years of follow up after diagnosis of AVN	10.11 (±6.18)
Years of total follow-up	37.95 (±17.79)

## Data Availability

The data that support the findings of this study are available from the corresponding author upon reasonable request.
